# Sphenopalatine Ganglion Block: Treatment of Migraine and Trigeminal Neuralgia Associated With Multiple Sclerosis

**DOI:** 10.7759/cureus.8522

**Published:** 2020-06-09

**Authors:** Michael Nagib, Preston Hood, Jerry Matteo

**Affiliations:** 1 Interventional Radiology, University of Florida College of Medicine, Jacksonville, USA; 2 Radiology, University of Florida College of Medicine, Jacksonville, USA

**Keywords:** trigeminal neuralgia, multiple sclerosis, sphenopalatine ganglion block, chronic pain, migraine

## Abstract

Head and facial pain are a burden to many people both directly and indirectly. This is manifested not only as a personal burden but also as a financial one in the form of sick leaves from work and loss of workplace productivity. These costs stem from emergency department visits, hospitalizations, preventative treatments, and medical management. Medical management of migraine headaches and other causes of facial pain often proves insufficient, and sphenopalatine ganglion block (SPGB) provides an innovative, adjunctive outpatient treatment option with excellent results in alleviating symptoms. We present a case of a young female suffering from headache and orofacial pain secondary to multiple sclerosis (MS) and trigeminal neuralgia (TN) refractory to traditional medical management, who underwent SPGB with immediate relief of her symptoms. Due to its effectiveness, the role of the SPGB in the treatment of various other conditions causing headache and orofacial pain, such as MS or even TN, continues to expand providing relief and restoring functionality.

## Introduction

When considering the alternative therapies for chronic migraine headaches and orofacial pain such as Gamma Knife radiotherapy, neuromodulation, or ablation, sphenopalatine ganglion block (SPGB) provides a valuable, minimally invasive approach, with a low incidence of adverse effects and the opportunity for repeat treatment as needed.

Multiple sclerosis (MS) is an autoimmune disease in which the body’s immune system attacks and destroys the protective coating of nerves called myelin. The effects of this destructive process are varied but can be manifested in the form of pain, loss of motor function or sensation, and vision loss. While for many the symptoms wax and wane, progressive forms do exist. In some patients, the diagnosis of trigeminal neuralgia (TN) often precedes the official diagnosis of MS [1]. In some studies, the percentage of patients who suffer from TN in addition to MS was approximately 10%. Additionally, approximately 15% of patients diagnosed with MS were first diagnosed with TN [1]. Again, many of these patients look to pharmacological agents such as anti-epileptic drugs, tricyclic antidepressants, and even opioids for symptom relief, whereas others seek more aggressive treatment measures in the form of Gamma Knife radiotherapy.

TN is a chronic pain condition, which can be episodic or constant in nature, characterized by orofacial pain that is sudden and shock-like, stabbing or burning in character. TN is a result of stimulation/irritation of the trigeminal nerve, or fifth cranial nerve, which supplies sensory information from the upper, middle, and lower thirds of the face and oral cavity to the brain [2]. Pain attacks may be precipitated by even routine stimulation of the areas of the face corresponding to the trigeminal nerve, such as in shaving, applying make-up, eating, or even wind exposure [2]. Trauma, surgery, or compression from an adjacent mass may cause irritation of this nerve and result in the debilitating symptoms of TN. Alternatively, disease processes that affect the integrity of the nervous system, such as MS, may also result in neuropathic facial pain. Currently, treatment of symptoms is typically managed pharmacologically, although more aggressive surgical options such as neurectomy or radiosurgery using Gamma Knife may be employed for refractory cases [2].

## Case presentation

Our patient is a 38-year-old Caucasian female who initially presented with intermittent lower extremity paresthesia and blurred vision beginning in 2002. At that time, cerebrospinal fluid analysis was negative; however, the patient continued to have an intermittent exacerbation of symptoms. Therefore, she was medically managed for symptoms related to MS. In 2015, she developed left orofacial pain, which was thought to be secondary to TN. She continued medical management of her symptoms using carbamazepine, gabapentin, trazodone, and baclofen. Despite medical management, the patient’s paresthesia, visual disturbances, and facial pain persisted. Of note, the patient also underwent physical therapy for bilateral lower extremity weakness, with mild improvement.

**Figure 1:**
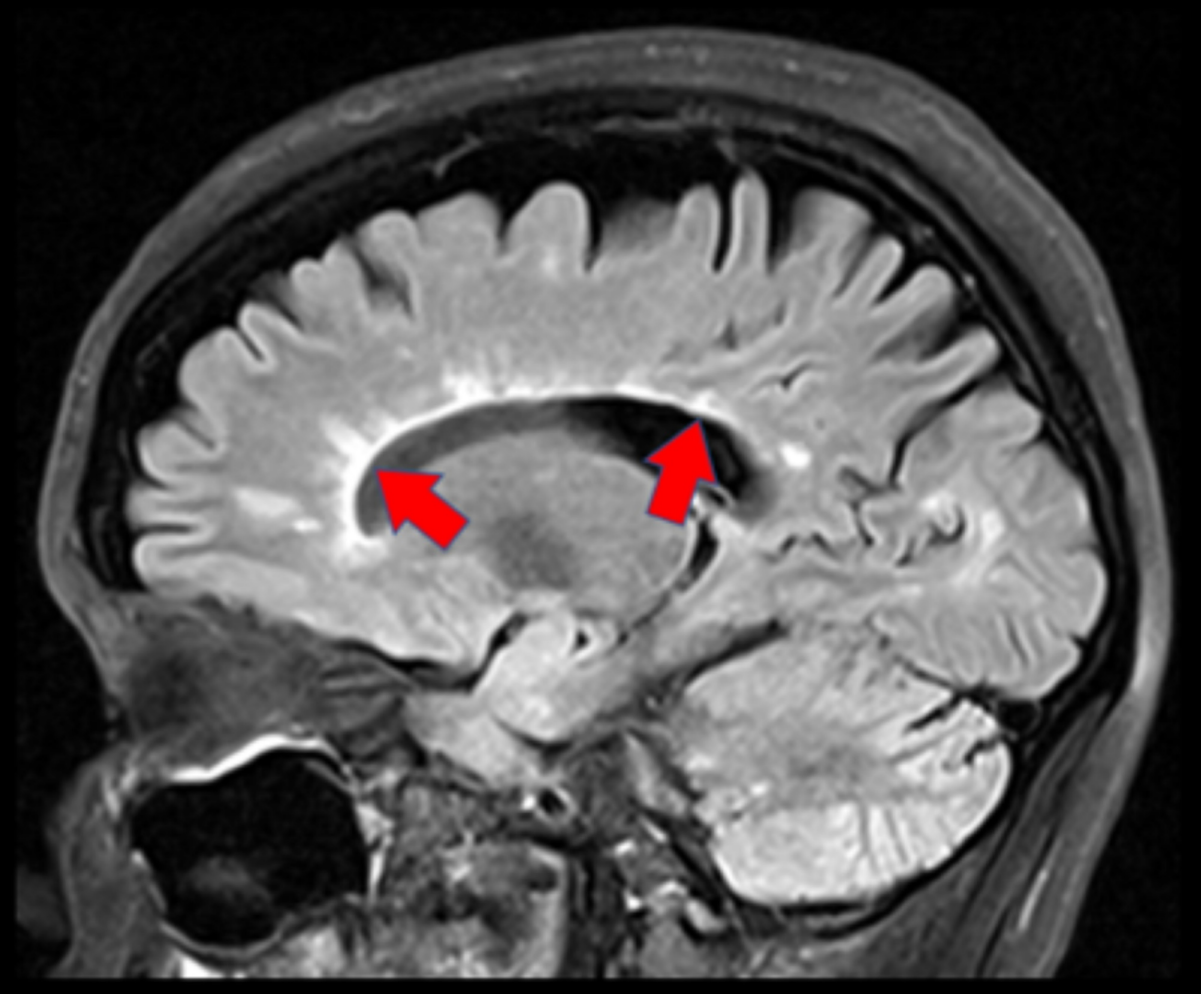
Sagittal view of the patient’s brain on MRI through the corpus callosum demonstrating multiple pericallosal finger-like projections of T2 hyperintensity representing demyelinating plaques known as Dawson fingers (red arrows).

Imaging performed in 2017 during a severe exacerbation of her symptoms demonstrated multiple active demyelinating lesions compatible with an MS flare-up (Figure 1).

At this point in time, the patient was started on dimethyl fumarate in an effort to decrease the frequency of episodic exacerbation of her symptoms. However, after approximately one month, the patient was forced to discontinue the drug due to gastrointestinal side effects. For a brief period of approximately two months in early 2018, the patient had no new or increasing symptoms.

In 2018, follow-up imaging was again performed, which demonstrated higher lesion burden than expected, and due to the patient’s history of positive John Cunningham virus antibody, she was started on ocrelizumab.

The patient subsequently developed new right-sided facial pain, right-sided vision loss, and ataxia with multiple falls, which required inpatient admission and a three-day course of steroids.

Due to the worsening of her symptoms, specifically her debilitating facial pain, the patient was referred to Interventional Radiology for SPGB. The patient was evaluated and elected to proceed with SPGB in an effort to relieve her long-standing facial pain.

**Figure 2:**
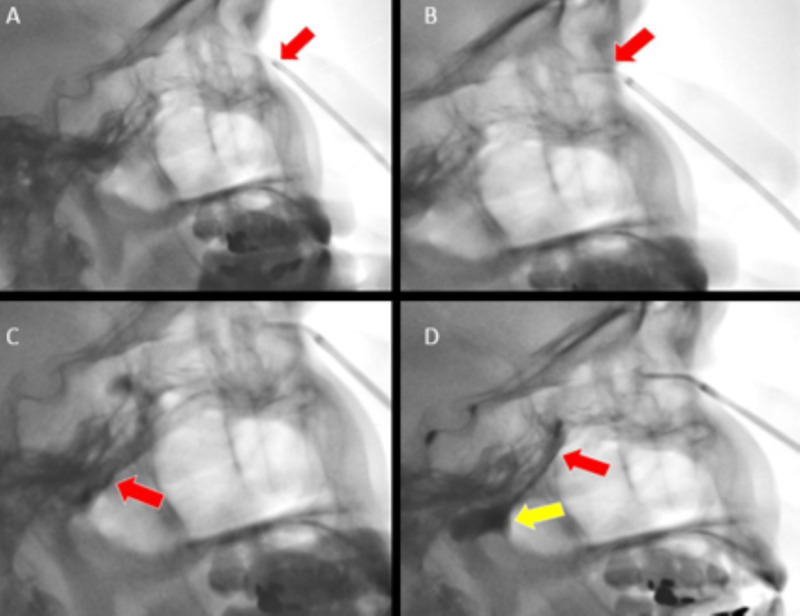
(A) Lateral fluoroscopic image shows the SphenoCath intranasal catheter in position with the inner catheter retracted (red arrow). (B) Lateral fluoroscopic image shows the SphenoCath intranasal catheter in position with the inner catheter now extended (red arrow) above and beyond the anterior border of the middle turbinate. (C) Lateral fluoroscopic image shows OmnipaqueTM contrast flowing through the pterygopalatine canal (red arrow). (D) Lateral fluoroscopic image demonstrates the Omnipaque flowing through the pterygopalatine canal (red arrow) and pooling of the contrast in the sphenoethmoid recess (yellow arrow).

The patient presented as an outpatient for SPGB. First, the patient was placed supine with a shoulder bump to maximize SPG accessibility. An atomizer was inserted into the nostril, and the patient inhaled through the nose as 2% lidocaine was injected for pre-procedure anesthesia. The SphenoCath® was lubricated with 2% lidocaine gel, and a catheter was placed into the nostril in a retracted position with the direction indicator facing anteriorly. Using fluoroscopic guidance, the catheter was inserted along the anterior portion of the nasal passage until it gently contacted the superior nasal bone (Figure 2A). It was then withdrawn 5 mm, and the inner catheter was advanced into the extended position (Figure 2B). Care was taken so that the lidocaine was injected above the middle turbinate and below the superior turbinate. Position was confirmed prior to treatment with contrast injection (Figure 2C). Contrast was seen flowing over the middle turbinate into the sphenopalatine fossa, creating a disc-shaped pooling of contrast and air-fluid level in this recess (Figure 2D). Once proper placement had been verified, catheter position was maintained, and 2 mL of 4% lidocaine was injected. The patient then maintained this position for approximately 10 minutes. The patient had immediate relief following the procedure. Six-week follow-up revealed that the patient was symptom-free and doing well at the time this article was submitted.

## Discussion

Annual medical expenditure related to migraine headaches in the United States reaches billions of dollars and results in over 100 million workdays lost per year [3]. Throughout the years, medical management has proven to be less than optimal in the treatment and prevention of migraines, and SPGB provides a safe and quick outpatient treatment option with excellent results in our patient population.

Hypersensitivity of the sphenopalatine ganglion (SPG) is suspected to be a key culprit in the etiology of migraine and orofacial pain symptoms. The SPG is a crossroad for neural pathways and houses sensory, sympathetic, and parasympathetic fibers in the pterygopalatine fossa posterior to the middle nasal turbinate. Afferent fibers from the trigeminal nerve initially synapse on the superior salivatory nucleus (SSN) [4,5]. The preganglionic parasympathetic fibers of the SSN are associated with postganglionic fibers of the SPG [5]. Postganglionic fibers of the SPG then provide innervation of the lacrimal, nasal, and pharyngeal glands [4,5].

The sensory roots of the SPG are derived from the maxillary nerve, a branch of the trigeminal nerve. From the SPG, small nerve roots project and transmit back sensory information from the midface, in addition to the orbit and oral cavity. It is these nerve roots that likely play a strong role in the transmission of parasympathetic outflow, causing headache and orofacial pain associated with migraines, TN, and neuropathic pain secondary to MS [5,6]. Disruption of the parasympathetic outflow at the SPG crossroad allows for a single target therapy for migraines, headaches, and multiple orofacial pain etiologies [5].

Review of our data regarding SPGB and positive patient outcomes demonstrates that SPGB is an effective, well-tolerated, and noninvasive means of managing head and facial pain of various etiologies. While efficacy does vary with the etiology of the pain being treated, many of our patients treated for migraine/headache reported rapid relief of pain that was maintained through the initial 24-hour post-procedural period. In addition, regarding the long-term efficacy of the SPGB, many of our patients have described symptom relief up to six months without the need for retreatment.

Adverse events related to SPGB through a transnasal approach tend to be minor in severity in the form of numbness of the throat, nasal pain, nausea, and dizziness [7]. In general, a minority of patients report no improvement of symptoms; however, a majority typically endorse at least mild, temporary relief from acute facial pain attacks and headaches.

## Conclusions

SPGB is an effective treatment for migraines and other etiologies of head and orofacial pain, including MS and TN, with minimal risk and a low complication profile. The nature of the SPGB, with its flexibility and procedural ease, offers a quick and effective single-target technique for head and facial pain associated with many conditions. Being able to offer a minimally invasive, practical, focused treatment for this patient population is beneficial not only to patients but also to employers and the healthcare system overall. Specifically, SPGB could benefit patients who have recalcitrant, debilitating TN and are considering surgical options.
